# Estimating the minimum important difference in the DEMQOL instrument in people with dementia

**DOI:** 10.1007/s11136-021-02900-7

**Published:** 2021-06-10

**Authors:** Ellen C. Lee, Jessica Wright, Stephen J. Walters, Cindy L. Cooper, Gail A. Mountain

**Affiliations:** 1 Sheffield Clinical Trials Research Unit, School of Health and Related Research, The University of Sheffield, Regent’s Court, 30 Regent St, Sheffield, S1 4DA UK; 2 Centre for Applied Dementia Studies, University of Bradford, Bradford, UK

**Keywords:** People with dementia, DEMQOL, DEMQOL-U, Minimum important difference, Responsiveness

## Abstract

**Purpose:**

The Dementia-Related Quality of Life (DEMQOL) measure and the DEMQOL-Utility Score (DEMQOL-U) are validated tools for measuring quality of life (QOL) in people with dementia. What score changes translate to a clinically significant impact on patients’ lives was unknown. This study establishes the minimal important differences (MID) for these two instruments.

**Methods:**

Anchor-based and distribution-based methods were used to estimate the MID scores from patients enrolled in a randomised controlled trial. For the anchor-based method, the global QOL (Q29) item from the DEMQOL was chosen as the anchor for DEMQOL and both Q29 and EQ-5D for DEMQOL-U. A one category difference in Q29, and a 0.07 point difference in EQ-5D score, were used to classify improvement and deterioration, and the MID scores were calculated for each category. These results were compared with scores obtained by the distribution-based methods.

**Results:**

A total of 490 people with dementia had baseline DEMQOL data, of these 386 had 8-month data, and 344 had 12-month DEMQOL data. The absolute change in DEMQOL for a combined 1-point increase or decrease in the Q29 anchor was 5.2 at 8 months and 6.0 at 12 months. For the DEMQOL-U, the average absolute change at 8 and 12 months was 0.032 and 0.046 for the Q29 anchor and 0.020 and 0.024 for EQ-5D anchor.

**Conclusion:**

We present MID scores for the DEMQOL and DEMQOL-U instruments obtained from a large cohort of patients with dementia. An anchored-based estimate of the MID for the DEMQOL is around 5 to 6 points; and 0.02 to 0.05 points for the DEMQOL-U. The results of this study can guide clinicians and researchers in the interpretation of these instruments comparisons between groups or within groups of people with dementia.

**Trial Registration Number and date of registration:**

ISRCTN17993825 on 11th October 2016.

**Supplementary Information:**

The online version contains supplementary material available at 10.1007/s11136-021-02900-7.

## Plain english summary

The Dementia-Related Quality of Life questionnaire is one way of measuring the quality of life of people diagnosed with dementia. The questionnaire has 29 questions, about topics including looking after yourself, health and well-being and relationships and scores range between 28 and 112. Higher scores indicate better quality of life. Until now, when using this questionnaire for research, we did not know what change or difference in scores a person with dementia would feel was important and which would cause doctors to think about a change in the person’s treatment or care. After using statistical techniques to assess this, we estimate that people with dementia would feel that a questionnaire score change of 5 or 6 points would be helpful and would cause their doctors to think about a change in the person’s treatment or care.

This information will help researchers to design future trials (they can work out how many people must be recruited) and analyse the results (has the activity or drug had a clinically significant effect on people?).

We also looked at the DEMQOL-Utility score (used by health economists to understand if activities or drugs are value for money) which ranges from 0.243 to 0.986 and is calculated using the Dementia-Related Quality of Life questionnaire scores. We found that a 0.02 to 0.05 points increase in the DEMQOL-Utility score would indicate that the person being tested was experiencing a clinically significant increase in quality of life.

## Introduction

Dementia is an umbrella term for a variety of diseases and conditions affecting the brain distinguished by a decline in memory, language, problem-solving and other thinking skills that affect a person's ability to perform everyday activities. There are a number of sub-types of dementia, but the five most common are Alzheimer’s disease, vascular dementia, dementia with Lewy bodies, frontotemporal dementia and mixed dementia. Alzheimer's is the most common cause of dementia [1]. As a person’s age increases, so does the risk of them developing dementia [2].

Worldwide, around 50 million people have dementia, and there are up to 10 million new cases every year [3]. Projections show that there will be 82 million people with dementia in 2030 and 152 million in 2050 [3]. Alzheimer’s Research UK reports that there are over 850,000 people currently living with dementia in the UK [4]. It is estimated that the number of people with dementia in the UK will rise to over one million by 2021 [1].

There is no treatment currently available to cure dementia or to alter its progressive course. Numerous new treatments are being investigated in various stages of clinical trials [3]. However, much can be offered to support and improve the lives of people with dementia and their careers and families and services are being encouraged to provide post-diagnostic treatment and support [5].

Given that we are currently unable to cure people with dementia, maintaining or enhancing quality of life (QOL) is often an important therapeutic goal [6]. Therefore, accurately measuring QOL is required to both guide clinical decision making and evaluate the impact of various interventions in the population of people with dementia. For this purpose, validated instruments have been developed to evaluate QOL in patients with dementia including the DEMQOL and DEMQOL-U. The DEMQOL is a patient-reported outcome measure (PROM) which is designed to enable the assessment of health-related quality of life of people with dementia [7]. The DEMQOL-U consists of items from the DEMQOL and generates a preference-based single index or score for use in economic evaluations [8].

Interpreting the numerical scores on these instruments can be challenging for a number of reasons. The scales and instruments used may be unfamiliar to many patients and clinicians, who may be uncertain of the meaning of the scale values and summary scores [9]. It is relatively simple to determine the statistical significance of a change in QOL but placing the magnitude of these changes in a context that is meaningful for patients, health professionals, and other stakeholders (pharmaceutical and medical device developers, insurance payers, regulators, governments) has not been so easy. Ascertaining the magnitude of change that corresponds to a minimal important difference would help to address this problem.

Jaeschke et al. 1989 defined the minimal important difference, from the patient perspective, as “The smallest difference in score in the domain of interest which patients perceive as beneficial and which would mandate, in the absence of troublesome side effects and excessive cost, a change in the patient’s management” [10].

Thus, individual change standards are needed to provide meaningful interpretation of treatment effects on QOL and to classify patients based on this standard as improved, stable or declined. Anchor-based and distribution-based methods can be used for estimating the MID in QOL instruments [9, 11, 12]. The aim of this study was to estimate the MID for the DEMQOL and DEMQOL-U instruments for comparisons between groups or within groups of people with dementia in a prospective cohort of people with dementia who were recruited as part of an RCT.

## Methods

### The journeying through dementia trial

The data for this study are provided from the Journeying through Dementia (JtD) Trial, a randomised controlled trial that recruited participants with early-stage dementia from across the UK [13]. In total, 490 participants completed baseline data, 480 of these participants were randomised 1:1 to receive usual care or the JtD programme plus usual care. Participants were eligible for the study if they had a diagnosis of dementia, a Mini Mental State Examination (MMSE) score of 18 or more (indicating that the person was in the milder stages of dementia), a good understanding of English, and were living in the community (not in residential or nursing care). The Journeying through Dementia programme involved twelve weekly group sessions with two trained health practitioners and four individual one-to-one sessions with one of those practitioners. The trial ran 28 groups across 13 sites and those registered for each group varied from four to 12. Participants were followed up at eight and 12 months, and the primary outcome was DEMQOL at eight months post-randomisation.

### Instruments and scoring

The DEMQOL questionnaire contains 29 questions [7]. The first 28 items are summed to calculate the DEMQOL score and cover 5 domains: daily activities and looking after yourself, health and well-being, cognitive functioning, social relationships and self-concept. It is completed by the person with dementia, in the case of JtD through interview in the person’s home. Each item in the DEMQOL is rated on a 4-point scale: 1 (a lot), 2 (quite a bit), 3 (a little) and 4 (not at all). A separate global quality of life item, Q29, asks respondents to rate ‘your quality of life overall’ in the past week on 4-point scale, which is rated 1 (very good), 2 (good), 3 (fair) and 4 (poor) (see Online Resource 1 for full wording of Q29). Question 29 does not contribute to the DEMQOL total score. Six items (including Q29) are reverse coded so that for all items, a higher score means better health-related quality of life. Responses to the 28 items on the DEMQOL are summed to generate a total score on a 28 to 112 scale, where higher scores indicate better health-related quality of life.

The DEMQOL-U classification system comprises five dimensions (positive emotion, memory, relationships, negative emotion and loneliness) with four levels of increasing impairment associated with each dimension [8]. The scoring algorithms for the DEMQOL-U were derived using the time trade-off elicitation technique in a UK general population sample. The resulting utility scores lie on the zero to one quality-adjusted life years (QALY) scale where zero represents the state dead and one represents the state of full health. The utility scores for the DEMQOL-U range from 0.243 to 0.986.

The EQ-5D-5L (hereinafter referred to as EQ-5D) is a health status classification measured over 5 dimensions: mobility, self-care, usual activities, pain/discomfort and anxiety/depression [14]. Each dimension is assessed by a single question on a five-point ordinal scale (e.g. no problems, slight problems, moderate problems, severe problems, unable). The EQ-5D preference-based measure can be calculated by assigning preference weights value sets to the raw scores. The EQ-5D preference-based score ranges from -0.59 to 1.00 where 1.00 indicates “full health”, 0.00 represents dead, and a negative score represents a health status valued as worse than dead.

### Statistical analysis

All statistical analyses were performed in Stata v16 [15]. The DEMQOL score was calculated by summing the response to the first 28 items when at least half (14 or more) of the items were answered, the missing items were imputed with the mean of the completed items [16]. The EQ-5D was scored using the mapping function developed by van Hout et al. [17], no score was calculated if any items were missing. Summaries and analysis were presented on JtD participants that had at least baseline data (*n* = 490), this is more than those randomised as a few participants withdrew prior to randomisation.

Both anchor-based and distribution-based methods were used to determine the MID as recommended by Revicki et al. [12]. For the anchor-based methods, the response to question 29 of the DEMQOL (DEMQOL Q29), which asks “… in the last week, how would you rate your quality of life overall” was considered as an anchor for DEMQOL as it is a self-reported single question rating of overall quality of life. The four responses to this item are very good, Good, Fair and Poor. The DEMQOL Q29 and EQ-5D were considered as anchors for DEMQOL-U. Spearman’s correlation coefficient was calculated to assess the correlation between the measures and the anchors with values over 0.3 deemed as having acceptable association between anchor and the outcome measure [12]. For the cross-sectional anchor analysis, baseline DEMQOL and DEMQOL-U were summarised over the four DEMQOL Q29 response categories (Quality of life overall: very good, good, fair and poor). One-way ANOVA was used to test for difference in baseline DEMQOL and DEMQOL-U means across the four Q29 response groups.

For the longitudinal anchor, change scores were calculated for each outcome for baseline to 8 months and baseline to 12 months. A change of 0.07 in EQ-5D score was used to classify a negative or a positive change; this was guided by an observed mean MID of 0.074 over 11 studies from a review by Walters and Brazier [9]. Participants with a less than 0.07 absolute change, in the EQ-5D score, were classified as no change. For the DEMQOL, change over time from baseline to follow-up was summarised over the seven possible Q29 change categories. The DEMQOL-U change was summarised over both Q29 and EQ-5D response categories. The negative and positive change scores were combined to provide a single MID estimate when the MID evidence was consolidated (for example, a 1 category improvement with the absolute values of a 1 category deterioration in Q29 scores) hence assuming the cohorts being combined are identical except for the sign.

All distribution-based methods are sample dependent (as they use sample SD) and are all considered as proxies to MID as they do not provide information about a difference that is considered minimally important [18]. Hence, they were used to provide supporting evidence of the MID and compared with the anchor-based MID scores. The previously observed effect size was reported, alongside SD per treatment group at baseline for studies that used DEMQOL. Studies using DEMQOL were identified through literature searches on Medline and EMBASE for randomised controlled trials reported in English in any population type. Studies solely using DEMQOL-Proxy, or a translated version of DEMQOL, were not included. The search also highlighted several observational studies using DEMQOL that were included in the results either due to their large scale or relevance.

The standardised effect size was calculated using the between-person SD at baseline from the JtD data for small (0.2 SD) to medium (0.5 SD) effect sizes [19]. Smith et al. [7] found the test–retest reliability (intra-class correlation) of DEMQOL to be 0.84 (*n* = 17) amongst the whole sample and 0.76 (*n* = 10) amongst the subset with mild/moderate MCI MMSE ≥ 10. Reliability (Internal consistency—Cronbach’s alpha) was found to be 0.87 (*n* = 75) amongst the whole sample and 0.87 (*n* = 68) amongst those with MMSE ≥ 10. The standard error of measurement (SEM [20]) was calculated using the between-person SD at baseline and scale reliability (*r)* observed by Smith et al. [7] ($\mathrm{SEM}=\mathrm{SD}\sqrt{\left(1-r\right)}$). The SEM was calculated using both test–retest reliability and Cronbach’s *α*.

## Results

A total of 490 participants were recruited to the JtD study and had available baseline DEMQOL data, of these 386 also had 8-month data, and 344 had both baseline and 12-month DEMQOL data. Table 1 summarises the characteristics of the cohort. The median age of the cohort was 78.0 and 58% of the participants were male. The most prevalent diagnosis was Alzheimer’s (60%), the median length of time since dementia was diagnosed was 0.7 years (IQR 0.3–1.8 years). The inclusion criteria for this study were a MMSE score of 18 or more, so all participants in the cohort had either mild cognitive impairment (38%) or normal cognitive function (62%) at baseline according to the MMSE. The characteristics are presented separately for those that had baseline data, baseline and 8-month data, and baseline and 12-month data, the three groups appear to have similar characteristics, with a slightly lower proportion of participants with mild cognitive impairment in the group with 12-month data available. Table 2 shows how the mean DEMQOL and DEMQOL-U scores changed over time for the whole cohort and the sub-sample who completed all three assessments. Overall, there was little change in DEMQOL and DEMQOL-U scores over time.

**Table 1:** Baseline characteristics for JtD participants with available data (*n* = 490)

Characteristic	With baseline data (*n* = 490)	With 8 m data (*n* = 386)	With 12 m data (*n* = 344)
Sex			
Male	283 (57.8%)	227 (58.8%)	206 (59.9%)
Female	207 (42.2%)	159 (41.2%)	138 (40.1%)
Age			
*n* (%)	490 (100.0%)	386 (100.0%)	344 (100.0%)
Mean (SD)	77.1 (7.4)	76.4 (7.5)	76.4 (7.4)
Median (IQR)	78.0 (73.0, 83.0)	77.0 (72.0, 82.0)	77.0 (72.0, 81.0)
Min., Max	39.0, 93.0	39.0, 93.0	39.0, 90.0
Ethnicity			
English/Welsh/Scottish/Northern Irish/British	468 (95.5%)	368 (95.3%)	325 (94.5%)
Irish	7 (1.4%)	5 (1.3%)	6 (1.7%)
Any other White background	5 (1.0%)	4 (1.0%)	4 (1.2%)
Indian	3 (0.6%)	3 (0.8%)	3 (0.9%)
Other	6 (1.2%)	6 (1.6%)	6 (1.7%)
Unknown	1 (0.2%)	0 (0.0%)	0 (0.0%)
Lives with others			
No	129 (26.3%)	92 (23.8%)	84 (24.4%)
Yes	360 (73.5%)	293 (75.9%)	259 (75.3%)
Type of dementia diagnosed			
Alzheimer’s	296 (60.4%)	242 (62.7%)	216 (62.8%)
Vascular dementia	50 (10.2%)	34 (8.8%)	29 (8.4%)
Mixed Alzheimer’s/vascular dementia	112 (22.9%)	85 (22.0%)	77 (22.4%)
Dementia in Parkinson disease	6 (1.2%)	5 (1.3%)	4 (1.2%)
Frontotemporal dementia (FTD)	7 (1.4%)	6 (1.6%)	5 (1.5%)
Lewy body dementia	4 (0.8%)	3 (0.8%)	3 (0.9%)
Unspecified dementia	12 (2.4%)	10 (2.6%)	9 (2.6%)
Other	2 (0.4%)	1 (0.3%)	1 (0.3%)
Length of time since dementia diagnosis (years)			
*n* (%)	479 (97.8%)	385 (99.7%)	343 (99.7%)
Mean (SD)	1.3 (1.6)	1.3 (1.6)	1.4 (1.7)
Median (IQR)	0.7 (0.3, 1.8)	0.8 (0.3, 1.8)	0.8 (0.3, 1.9)
Min., Max	0.0, 13.0	0.0, 13.0	0.0, 13.0
MMSE cognitive impairment			
Mild	184 (37.6%)	128 (33.2%)	113 (32.8%)
Normal	306 (62.4%)	258 (66.8%)	231 (67.2%)
MMSE (total score)			
*n* (%)	490 (100.0%)	386 (100.0%)	344 (100.0%)
Mean (SD)	24.6 (3.1)	24.9 (3.1)	25.0 (3.1)
Median (IQR)	25.0 (22.0, 27.0)	25.0 (23.0, 27.0)	25.0 (23.0, 28.0)
Min., Max	18.0, 30.0	18.0, 30.0	18.0, 30.0

**Table 2:** DEMQOL and DEMQOL-U summaries by timepoint for JtD participants (N = 490)

	DEMQOL			DEMQOL-U		
	*n*	Mean	SD	n	mean	SD
Participants with available data						
Baseline	490	90.7	13.0	490	0.862	0.114
8 months	388	92.6	13.8	386	0.87	0.120
12 months	352	92.0	14.1	345	0.872	0.117
Participants with data at all three timepoints						
Baseline	337	90.9	13.4	337	0.865	0.118
8 months	337	92.2	14.1	337	0.868	0.124
12 months	337	92.1	14.0	337	0.872	0.117

All correlations between DEMQOL(-U) and the anchors at each timepoint are above the threshold of 0.3 and so the anchors have an appreciable association with the DEMQOL and DEMQOL-U (see Online Resource 2). DEMQOL-U has slightly higher correlation with Q29 than it does with EQ-5D. The correlation between change scores at 8 and 12 months for DEMQOL-U with both EQ-5D and Q29 was below the threshold of 0.3; EQ-5D was particularly low so the longitudinal anchor analysis for DEMQOL-U and EQ-5D is to be treated cautiously.

### Anchor-based methods

The distributions of DEMQOL and DEMQOL-U at baseline over the four Q29 quality of life overall categories are displayed in Table 3. The mean DEMQOL score is eight points higher in the participants that chose good health compared to fair, and the difference is similar in those that chose very good compared to good. The difference in DEMQOL-U between categories was around 0.06 (0.064 between fair and good, 0.057 between good and very good). The one-way ANOVA for the difference in means across the four categories was statistically significant (*p* < 0.001) for both DEMQOL and DEMQOL-U.

**Table 3:** DEMQOL and DEMQOL-U summaries at baseline, across DEMQOL Q29 categories (cross-sectional anchor)

Baseline	*n*	Mean	SD	95% CI for mean
DEMQOL				
Q29 Response				
Poor	10	61.6	16.1	50.5–73.2
Fair	89	81.8	11.9	79.3–84.3
Good	239	89.8	11.3	88.3–91.2
Very good	152	99.2	8.3	97.9–100.5
DEMQOL-U				
Q29 Response				
Poor	10	0.544	0.197	0.404–0.685
Fair	89	0.799	0.117	0.774–0.823
Good	239	0.863	0.092	0.851–0.875
Very good	152	0.920	0.080	0.907–0.933

ANOVA *p* < 0.001

The longitudinal anchor analysis is presented in Table 4. A one category increase in DEMQOL Q29 corresponds with a 7.2 average increase at 8 months and a 7.7 increase at 12 months in DEMQOL score. The absolute difference in DEMQOL is smaller for a one category decrease in Q29 at both 8 months and 12 months (− 3.2 change at 8 months and − 4.3 change at 12 months). Combining the two categories results in an average 5.2 point absolute change at 8 months and 6.0 point change at 12 months. The absolute change in DEMQOL-U for a 1-point increase or 1-point decrease in Q29 ranges from 0.023 to 0.054, and DEMQOL-U change related to a positive or negative change in EQ-5D ranges from 0.007 to 0.027 in absolute value. The absolute values of the mild improvement and deterioration groups were combined; these resulted in an average absolute change at 8 and 12 months being 0.032 and 0.046 for the Q29 anchor and 0.020 and 0.024 for EQ-5D anchor.

**Table 4:** DEMQOL and DEMQOL-U change by DEMQOL Q29 and EQ-5D change categories (longitudinal anchor)

	*n*	Mean	SD	95% CI for mean
DEMQOL change Baseline to 8 months				
Q29 change				
− 3	3	− 22.8	18.6	− 69.1–23.5
− 2	6	− 4.2	10.3	− 15.0–6.7
− 1	80	− 3.2	9.6	− 5.3 to − 1.0
0	210	1.6	9.1	0.3–2.8
1	81	7.2	9.8	5.0–9.4
2	7	11.4	10.6	1.6–21.3
3	1	35.0		
DEMQOL change Baseline to 12 months				
Q29 change				
− 2	12	− 8.8	10.3	− 15.3–− 2.3
− 1	66	− 4.3	10.1	− 6.7 to − 1.8
0	199	1.3	9.0	0.0–2.5
1	69	7.7	9.6	5.4–10.0
2	3	12.7	4.0	2.6–22.7
DEMQOL− U change Baseline to 8 months				
Q29 change				
− 3	2	− 0.063	0.262	− 2.420–2.295
− 2	6	− 0.075	0.066	− 0.145 to − 0.006
− 1	80	− 0.023	0.109	− 0.047–0.001
0	209	0.002	0.114	− 0.013–0.018
1	81	0.041	0.090	0.021–0.061
2	7	0.078	0.071	0.012–0.144
3	1	0.545		
EQ-5D change				
Negative*	94	− 0.007	0.106	− 0.028–0.015
No change*	165	0.007	0.101	− 0.008–0.023
Positive*	124	0.017	0.131	− 0.006–0.040
DEMQOL-U change Baseline to 12 months				
Q29 change				
− 2	12	− 0.045	0.085	− 0.099–0.009
− 1	65	− 0.039	0.096	− 0.063 to − 0.015
0	196	− 0.005	0.093	− 0.008–0.018
1	68	0.054	0.114	0.026–0.081
2	3	0.139	0.102	− 0.033–0.311
EQ-5D change				
Negative*	87	− 0.017	0.104	− 0.040–0.005
No change*	140	0.006	0.103	− 0.011–0.023
Positive*	113	0.027	0.096	0.009–0.045

*Negative change defined as ≤ 0.07, no change defined as between − 0.07 and 0.07, positive change defined as > 0.07

### Distribution based methods

#### Previously observed effect size

The JtD trial compared DEMQOL outcome between the treatment groups at 8 or 12 months post-randomisation. In a review of other randomised controlled trials that used DEMQOL, we found 10 RCTs using DEMQOL as an outcome: eight of these had published results [21–28] and two were ongoing [29, 30]. Of the eight RCTs with published results, one used DEMQOL as the primary outcome [27]. Table 5 shows that one trial observed a statistically significant treatment difference of 7.4; however, the sample size was small and the standard deviations observed were not in line with the other studies [27]. Most of the observed standard deviations at baseline are between 12 and 15 which are in keeping with our observed SD of 13.1 across treatment groups.

**Table 5:** Standard deviation and observed treatment difference for studies using DEMQOL

Study	Observed *n* with outcome data (total)^a^	SD per group at baseline	Observed absolute treatment differences
Trials			
Journeying through Dementia	381	13.0, 13.2	Not yet published
HTA SADD trial^b^ [21]	456, 405	14.3, 12.8, 17.2	0.03–1.76
The CSP-RYCT trial [22]	138	12.4, 13.4, 11.2	0.173–2.54
SMILE study [23]	371, 343	15.5, 13.8	0.1, 0.05
Individual cognitive stimulation therapy for dementia [24]	227, 264	11.76, 13.55	0.33, 1.73
Maintenance cognitive stimulation therapy [25]	218, 199	10.9, 11.7	0.3, 0.86
The GREAT trial [26]	445, 417	12.9, 11.75	0.24, 1.08
Crotty et al. hip fracture [27]	90, 70	27.8, 8.48	0.3–7.4
NIHR TEAM study [28]	222	11.9, 13.4	0.7
Observational studies			
Park et al. 2017 [31]	785	12.5	n/a
Banerjee 2006^c^ [32]	101	13.75	n/a

^a^numbers separated by a comma are n at first follow-up and *n* at second follow-up

^b^DEMQOL data completion unknown, *n* is any outcome data

^c^*n* in cohort

#### Standardised effect size

Cohen [19] defined a 0.2 standardised effect size as small and 0.5 as medium. Using the between-person SD at baseline, of 13 points, a small standardised effect size in DEMQOL is 2.6 points and a medium 6.5 points (Table 6).

**Table 6:** Standardised effect sizes using a baseline SD of 13 points

	DEMQOL	DEMQOL-U
0.2 SD	2.6	0.023
0.3 SD	3.9	0.034
0.4 SD	5.2	0.046
0.5 SD	6.5	0.057

#### Standard error of measurement

Using the estimates of reliability for participants with MMSE ≥ 10, the SEMs in Table 7 are calculated; 6.37 for test-retest reliability and 4.69 for internal consistency.

**Table 7:** SEM calculations

SD	*r*	SEM: SD × $\sqrt{\left(1-r\right)}$
13	0.76 (Test–retest reliability)	6.37
13	0.87 (Internal consistency)	4.69

### Combining estimates

**Fig. 1:**
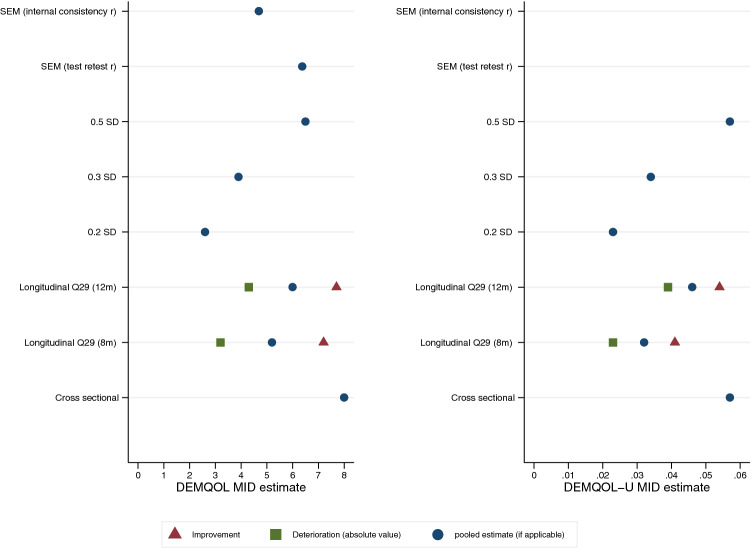
Summary of anchor and distribution-based Minimally Important Difference (MID) estimates for DEMQOL and DEMQOL-U method

Figure 1 shows the distribution and anchor-based estimates of MID for both DEMQOL and DEMQOL-U. The longitudinal anchor-based methods for improvement and deterioration are presented alongside the absolute change pooled for both improvement and deterioration groups. A MID of 5 is consistent with the longitudinal anchor MID estimate and is centred amongst the distribution-based methods, it corresponds to a 0.38 standardised effect size. EQ-5D anchor-based methods for DEMQOL-U have been excluded from the summary (Fig. 1); due to the low correlation between change EQ-5D and DEMQOL-U change scores, the estimates will be unreliable and likely to underestimate MID. The DEMQOL Q29-based anchor methods for DEMQOL-U fall in line with 0.3–0.5 SD estimate range.

## Discussion

We have calculated distribution, anchor, and standard error of measurement-based estimates of the MID for the DEMQOL and DEMQOL-U instruments using a large cohort of 490 patients with dementia. We found that the anchored-based estimate of the MID for the DEMQOL is around 5–6 points; and it is 0.02–0.05 points for the DEMQOL-U for comparisons between groups or within groups of people with dementia.

The minimum detectable change (MDC) has been defined as the smallest change in score (at an individual level) that can be detected after allowing for measurement error of the instrument. There are several methods for estimating the MDC, usually involving the standard error of measurement (SEM) calculated from reliability coefficients such as test–retest and Chronbach’s alpha [11]. We used two methods two estimate the SEM (test–retest reliability and internal consistency) and calculated values of 6.4 and 4.7 for one SEM, respectively, from these methods.

Ideally, for a sensitive and reliable instrument, the MDC should be smaller than the MID [11]. The MID and MDC are important but they are different concepts measuring different things. The relationship between the MID and MDC is discussed by Turner et al. 2010 and de Vet (2010) [33, 34]. They agree that the MDC (and the associated SEM) cannot reliability replace the MID. They conclude that the MDC is a statistical property of the measurement, but the MID is the value of concern for interpretation and is based on the judgement of patients. The MDC (and the associated SEM) is of little relevance for interpretation [11].

We observed that the MID value from the anchor-based assessment of 5 was smaller than the SEM of 6.4 (based on test–retest reliability). In these circumstances, changes as large as the MID may be important for patients, but they cannot be distinguished from measurement error [34]. We also observed that the MID of 5 was slightly larger the then SEM of 4.7 (based on internal consistency reliability). In this situation, changes as large as the MID can be considered statistically significant and important to patients [34].

To our knowledge, this study is the first to elicit and make recommendations on the MID for DEMQOL and DEMQOL-U. Both anchor and distribution-based methods were used, and results were consolidated. Anchor-based analysis was only conducted for anchors that achieved moderate correlation with the measure of choice (|*r*|≥ 0.3). Using EQ-5D as a longitudinal anchor may have over-estimated the MID estimate of DEMQOL-U as it included participants with a large absolute change in the estimate—however, the estimates that were observed were smaller than using DEMQOL Q29, this was likely due to the low correlation between change scores, producing unreliable, and underestimated MID estimates.

Using global QOL as an anchor is one accepted method and has been used in previous studies [9, 35, 36]. The advantages of using a global rating of QOL as an anchor are that they are relatively easy to obtain, patient-centred and can take into account a variety of information and determinants of well-being. However, in order to use an anchor-based method, there must exist some association (minimum correlation) between the QOL items and the chosen anchor [12]. A potential limitation of our study is that some of the correlations between the anchors used in our study, and the DEMQOL and DEMQOL-U scores were not that strong (the correlations ranged from 0.38 to 0.59), with the possible result that some of the anchors may not perform well in defining the MID. Nonetheless, these correlations were above the recommended minimum thresholds of 0.30 and the even higher value of 0.371 recommended by Hays et al. [37].

Other potential limitations associated with using DEMQOL Q29 as an anchor include recall bias and response shift [11]. Response shift occurs when a patient’s views, values or expectations change over time. Thus, a patient’s health might be seen to be getting worse, yet the patient with dementia may assert that their QOL has not changed, or even that it has improved. Alternatively, a patient’s health status may appear to be unchanging even though that same patient may report substantial changes in their QOL. It is difficult to assess response shift in people with dementia. The classical way of assessing response shift is to use the “then test” which may not be appropriate for patients with dementia. The then-test usually asked patients after completing a second assessment a question such as “We would like you to think back to the time of your first assessment. With hindsight, how would you now rate the way you felt then?” [11]. The difference between the values of the 1st assessment and the then-test provides an estimate of response shift.

Recall bias occurs when the respondent’s answer to a question is affected by the respondent’s memory. People tend to forget how extreme the past was. Response is likely to be influenced by the patient’s status at the time of recall. For these reasons, items with short recall periods or items that ask patients to describe their current or recent status are preferable [11]. The DEMQOL asks respondents questions about how they feel “right now” or “in the last week” so has a short recall period. However, response shift and recall bias remain a possibility with people with dementia, and this is a limitation. Indeed, given our patient population, our study is more likely to suffer from these possible threats to the validity of the results than other studies using similar anchor-based approaches.

We used a four-point global QOL anchor which resulted in seven-point change scale; others have used 14 points, which may be more sensitive [10]. The designation of what response on the DEMQOL Q29 anchor which suggests patients as fundamentally unchanged and what response suggests patients have experienced a small but important change is inevitably subjective.

The anchor, DEMQOL Q29, did not ask the participant to recall their overall health but instead rate it considering the previous week; this is a strength of the anchor and the global rating of QoL. However, a limitation of the study is that people with dementia had to self-report their quality of life and be able to do so again 12 months later, in which time their dementia may have progressed to moderate stages, and therefore, self-report could be less reliable for some participants. Only 70% (344/490) of participants completed the 12-month assessment; some of these may have been due to declining health.

This study was conducted in participants with mild/moderate dementia and in the relatively early stages of the disease, living in the community in the United Kingdom. Many (62%) of the participants were assessed as having normal cognitive function on the MMSE at baseline. The recommended MID estimates are most applicable to studies in a similar patient population—a single MID is unlikely to be relevant for all populations, and a different MID may be appropriate for later-stage dementia and participants living in residential or nursing care. A limitation of the study is that 21% of the participants were excluded from the longitudinal anchor analysis due to having missing outcome data (either having withdrawn from the study, lost to follow-up or death); however, Table 1 shows baseline characteristics to be similar for the subset that had available eight- and twelve-month data compared to all those that had baseline data.

Second, the follow-up duration was only 12 months, so the MID values that were determined herein may not be consistent with those in studies with longer follow-up durations. Thus, to validate the findings of this study, a longer follow-up and patients with different severities of dementia would be needed in future studies.

Our recommended MID should be confirmed based on evidence from other studies, and we recommend that other cohorts are used to estimate MID and the evidence is consolidated to provide a more robust estimate. Further research into the MID for the DEMQOL-proxy is needed for people with dementia who cannot self-report.

### Conclusion

In this study, we established MID scores for DEMQOL and DEMQOL-U using both anchor and distribution-based methods on a prospective cohort of people living with dementia. We found that the anchor-based estimate of the MID for the DEMQOL is around 5–6 points; and 0.02–0.05 for the DEMQOL-U. This study can guide researchers when designing studies and calculating sample sizes and when interpreting DEMQOL(-U) change over time and treatment differences for comparisons between groups or within groups of people with dementia.

## Supplementary Information

Below is the link to the electronic supplementary material.

Supplementary file1 (DOCX 13 kb)

Supplementary file2 (DOCX 14 kb)

## Data Availability

All data requests should be submitted to the corresponding author for consideration. Access to anonymised data may be granted following review.

## Code availability

All code requests should be submitted to the corresponding author for consideration.
