# Supplementary material for: Estimating the minimum important difference in the DEMQOL instrument in people with dementia
Source: Qual Life Res. 2021 Jun 10;30(10):2995–3005. doi: 10.1007/s11136-021-02900-7 (PMC8481142; doi:10.1007/s11136-021-02900-7)
Supplement: Supplementary file 1 — Supplementary file1 (DOCX 13 kb) [file 11136_2021_2900_MOESM1_ESM.docx]

# Online Resource 1

DEMQOL Q29 Wording

| **We’ve already talked about lots of things: your feelings, memory and everyday life. Thinking about all of these**  **things in the last week, how would you rate……….** | | Very good | Good | Fair | Poor |
| --- | --- | --- | --- | --- | --- |
| 29 | **your quality of life overall? **** | 4 | 3 | 2 | 1 |
