# Supplementary material for: Estimating the minimum important difference in the DEMQOL instrument in people with dementia
Source: Qual Life Res. 2021 Jun 10;30(10):2995–3005. doi: 10.1007/s11136-021-02900-7 (PMC8481142; doi:10.1007/s11136-021-02900-7)
Supplement: Supplementary file 2 — Supplementary file2 (DOCX 14 kb) [file 11136_2021_2900_MOESM2_ESM.docx]

# Online Resource 2

Table 8: Correlations between DEMQOL(-U), DEMQOL Q29 and EQ-5D

| Correlations |  |  |  |  |
| --- | --- | --- | --- | --- |
| DEMQOL and Q29 |  |  | DEMQOL and Q29 change from baseline | |
| All time points combined | 0.55 |  |  |  |
| Baseline | 0.53 |  |  |  |
| 8 months | 0.59 |  | 0.38 |  |
| 12 months | 0.54 |  | 0.40 |  |
|  |  |  |  |  |
|  |  |  | Change from Baseline | |
| DEMQOL-U… | …and Q29 | …and EQ-5D | …and Q29 | …and EQ-5D |
| All time points combined | 0.52 | 0.44 |  |  |
| Baseline | 0.49 | 0.44 |  |  |
| 8 months | 0.55 | 0.38 | 0.25 | 0.09 |
| 12 months | 0.51 | 0.50 | 0.31 | 0.17 |
|  |  |  |  |  |
